# Inverted terminal repeats of adeno-associated virus decrease random integration of a gene targeting fragment in *Saccharomyces cerevisiae*

**DOI:** 10.1186/1471-2199-15-5

**Published:** 2014-02-13

**Authors:** Alvaro Galli, Tiziana Cervelli

**Affiliations:** 1 Yeast Genetics and Genomics Group, Institute of Clinical Physiology, CNR, via Moruzzi 1, 56125 Pisa, Italy

**Keywords:** Yeast, AAV, ITRs, Homologous recombination, Random integration

## Abstract

**Background:**

Homologous recombination mediated gene targeting is still too inefficient to be applied extensively in genomics and gene therapy. Although sequence-specific nucleases could greatly stimulate gene targeting efficiency, the off-target cleavage sites of these nucleases highlighted the risk of this strategy. Adeno-associated virus (AAV)-based vectors are used for specific gene knockouts, since several studies indicate that these vectors are able to induce site-specific genome alterations at high frequency. Since each targeted event is accompanied by at least ten random integration events, increasing our knowledge regarding the mechanisms behind these events is necessary in order to understand the potential of AAV-mediated gene targeting for therapy application. Moreover, the role of AAV regulatory proteins (Rep) and inverted terminal repeated sequences (ITRs) in random and homologous integration is not completely known. In this study, we used the yeast *Saccharomyces cerevisiae* as a genetic model system to evaluate whether the presence of ITRs in the integrating plasmid has an effect on gene targeting and random integration.

**Results:**

We have shown that the presence of ITRs flanking a gene targeting vector containing homology to its genomic target decreased the frequency of random integration, leading to an increase in the gene targeting/random integration ratio. On the other hand, the expression of Rep proteins, which produce a nick in the ITR, significantly increased non-homologous integration of a DNA fragment sharing no homology to the genome, but had no effect on gene targeting or random integration when the DNA fragment shared homology with the genome. Molecular analysis showed that ITRs are frequently conserved in the random integrants, and that they induce rearrangements.

**Conclusions:**

Our results indicate that ITRs may be a useful tool for decreasing random integration, and consequently favor homologous gene targeting.

## Background

Gene targeting is the process by which exogenously delivered DNA is used to modify a genomic target by homologous recombination. Since in human cells the spontaneous frequency of homologous recombination is on the order of 10^-6^, the gene targeting approach is not efficient enough to be applied for clinical use [1]. However, several reports indicate that sequence-specific nucleases, which induce a site-specific double strand break in the target DNA, can increase gene targeting efficiency up to 50,000-fold [2]; this approach is thus particularly useful for manipulating primary human cells with therapeutic potential [3,4]. Although there has been considerable excitement about the potential application of zinc finger nucleases, since these enzymes allow highly specific, targeted genome modification in live cells [5], several reports show the off-target cleavage sites of these nucleases, highlighting the risk of using this approach [6,7]. Therefore, the search for new tools to improve the efficiency of gene targeting is still very important. Recently, the induction of a site-specific single-strand nick significantly increased gene targeting [8]. Our previously published studies show that the overexpression or nuclear permeation of the *Saccharomyces cerevisiae* protein Rad52 strongly increases homologous recombination and gene targeting in HeLa cells [9,10]. An alternative method of homologous recombination mediated gene targeting consists of exploiting the recombinogenic nature of the adeno-associated virus (AAV) vector genome [11]. The AAV single stranded (ss) DNA genome contains two overlapping open reading frames flanked by two inverted repeated sequences (ITRs), the only elements required in *cis* for replication and integration. The Rep open reading frame codes for Rep proteins essential for DNA replication, integration and packaging. The Cap codes for the proteins essential for capsid formation [12]. Vectors based on AAV, which deliver single-stranded, linear DNA genomes, are able to efficiently introduce many types of mutations into homologous target loci at a frequency approaching 1% in mammalian cells, and are currently used as gene targeting vectors [13-16]. However, using this method, each homologous targeted event occurs within ten random integrations [14]. Recently, by combining AAV technology with zinc finger nucleases, the efficiency of gene targeting increases up to 6% but most integration events still occur outside the target locus, most likely in naturally occurring DNA double-strand breaks [7,15-18].

Moreover, AAV-mediated gene targeting has been reported to be less dependent on the extent of homology between the vector and the genome target than other methods [14]. Notably, the presence of the ITRs flanking the gene targeting construct determines increased homologous recombination frequencies [19].

When AAV Rep proteins are expressed, the yeast *Saccharomyces cerevisiae* is able to replicate the ssDNA genome [20]. The proteins Rep68 and Rep40 are necessary for AAV replication and for site-specific integration [21,22]. Rep proteins interact with the Rep-binding element and the terminal resolution site sequences located within the ITRs, to create a nick that may increase the integration [23,24].

In the yeast *Saccharomyces cerevisiae*, gene targeting is very efficient; it occurs at a frequency of 2–3% and can be increased up to 25%, whereas random or non-homologous integration is reported to be less than 0.1% [25,26]. Yeast is an excellent genetic model for understanding the mechanisms and pathways involved in homologous and non-homologous recombination [27-30]. Moreover, yeast has been recently used as a system for studying the potential of a new genome editing approach for site-specific mutagenic and multiple allele replacement [31].

We decided to use the yeast *Saccharomyces cerevisiae* to evaluate whether the presence of ITRs in the integrating plasmid and the expression of AAV Rep proteins have an effect on gene targeting and random integration. The aim of this study was to assess whether the use of AAV sequences or expression of Rep proteins could be a feasible and valuable tool for increasing gene targeting or decreasing random integration.

## Results and discussion

### The presence of ITRs decreased the random integration of a gene targeting construct

**Figure 1:**
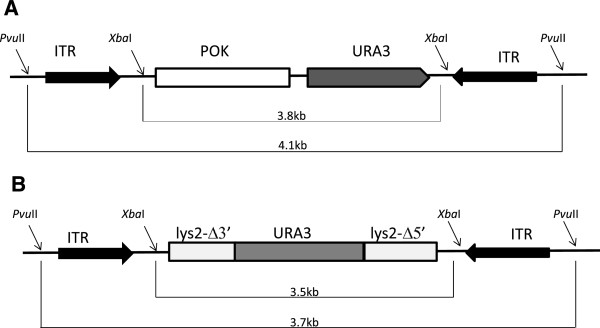
**Schematic representations of plasmids carrying the recombinant AAV fragment (rAAV). A)** pAAVpokURA. **B)** pAAVLUL. In both plasmids, restriction with *Pvu*II gives rise to the rAAV fragment containing the ITRs; restriction with *Xba*I cuts out the ITRs and generates a fragment with no ITR at both ends.

AAV vectors are often used for gene targeting experiments in mammalian cells also in combination with zinc finger technology [15,32,33]. Several studies indicate that AAV mediated gene targeting is affected by homologous recombination genes and that the AAV integration can be dependent on non-homologous end joining [34-37]; however, to our knowledge no comparative study has been performed to understand the role of ITRs and Rep proteins in gene targeting and random integration. Thus, to study the effect of ITRs on yeast gene targeting, we constructed a novel vector called pAAVLUL, containing the *LYS2* gene interrupted by *URA3* gene and flanked by the ITRs (Figure 1B). As shown in Figure 1B, the gene targeting fragment from the pAAVLUL was generated by two different restriction enzymes in order to keep the ITRs flanking both ends of the fragment, or not.

**Figure 2:**
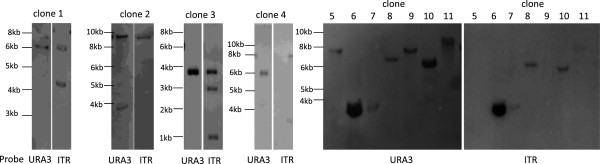
**Molecular analysis of random integration clones derived from transformation with ITRs carrying fragment.** Southern blot analysis of genomic DNA isolated from URA3^+^LYS2^+^ yeast transformant clones. These clones were obtained by transforming RSY12 yeast strain with the ITRs-containing fragment obtained by the digestion of pAAVLUL vector with *Pvu*II. We analyzed genomic DNA digested with *Ase*I of eleven different clones. The numbers above the filters indicate the clones. Bands were detected using the URA3 probe and the ITR probe as indicated.

**Figure 3:**
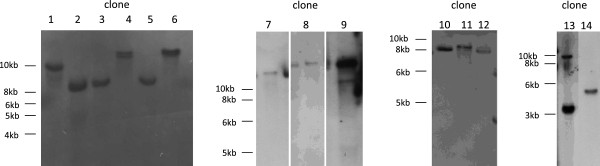
**Molecular analysis of random integration clones derived from transformation with no**-**ITRs**-**containing fragment.** Southern blot analysis of genomic DNA from fourteen URA3^+^LYS2^+^ yeast clones derived from transformation of RSY12 yeast strain with pAAVLUL digested with *Xba*I. Digestion with *Xba*I produces a fragment without ITR as described in Figure 1B. Genomic DNA was digested with *Ase*I that does not cut in the sequence between the ITRs. The numbers above the filters indicate the clones. Bands were detected using the URA3 probe.

Overall, gene targeting was not affected by the presence of ITRs (Table 1); however, the random integration increased by almost threefold when ITRs were not present (Table 1). Thus, when ITRs are present, the GT/RI ratio increased fifty fold because the random integration decreased (Table 1). Southern blot analysis of genomic DNA of eleven URA3^+^LYS2^+^ random integration clones derived from yeast transformed with ITRs-carrying fragments indicated that ten clones have a single URA3 integration (Figure 2; clones l, 3, 4, 5, 6, 7, 8, 9, 10, 11). Although the size of the band is higher than the size of the AAV construct (3.7 kb) (Figure 1B), three clones out of ten have no ITRs as detected by the hybridization of the blot with ITR probe (Figure 2; clone 4, 5 and 9). Moreover, three clones have two or more copies of the construct, one detected with URA probe (Figure 2, clone 2) and two detected with ITR probe (Figure 2, clone 1 and 3). Clone 2 integrated two copies of the DNA fragment, but only one copy contained ITRs. We considered “rearrangements” those bands which were detected by only one probe (URA3 or ITR) or those with a size smaller than rAAV (3.7 kb, Figure 1B). We can conclude that in six out of eleven clones (54%) some rearrangement occurred. In order to better understand whether these rearrangements are due to ITRs, we analyzed the genomic DNA of fourteen URA3^+^LYS2^+^ random integration clones derived from the transformation with no ITR carrying-fragment. Figure 3 shows that nine out of fourteen clones (64%) contained a single copy of the fragment (Figure 3, clones 2, 3, 5, 6, 7, 8, 10, 12, 14) and there was no band lower than 3.5 kb (the size of the fragment without ITRs, Figure 1B). To make sure that ITRs are really not present in the genomic DNA, we hybridized the filter with the ITR probe. No bands were detected (data not shown). This result suggests that no rearrangement has occurred in absence of ITRs. Finally, we sequenced the junctions in order to see where the gene targeting construct was randomly integrated. By using two primers starting from the two portions of *LYS2* gene in the fragment, we were able to sequence four junctions; three junction sites were located on chromosome II right next to the *LYS2* locus and one junction was on chromosome XVI. The precise analysis of the sequence did not reveal any preferred junction site. However, it is possible that the homology between the fragment and the genome drives non-homologous integration. Our results clearly indicate that the presence of ITRs flanking the homologous sequence in the gene targeting fragment decreased random integration in yeast but determined a higher number of rearrangements.

**Table 1:** Effect of the AAV ITR sequence on gene targeting and random integration in yeast

**Plasmid**	**Gene targeting**	**Random integration**	**GT/RI**
**ITR**-*lys2URA3lys2*-**ITR**	31.0 ± 11.3 (7025)	0.62 ± 0.37 (487)	50.2
*lys2URA3lys2*	35.4 ± 10.4 (6960)	1.82 ± 0.41 (3420)*	19.4

Yeast was transformed with *Pvu*II- or *Xba*I-restricted pAAVLUL as reported in Materials and Methods. Gene targeting and random integration events are reported as number of *URA3*^*+*^*lys2*^*-*^colonies /10^3^ transformants per μg of plasmid DNA and *URA3*^*+*^*LYS2*^*+*^colonies /10^3^ transformants per μg of plasmid DNA, respectively. The number of transformants per μg of DNA was determined by transformation with 1 μg of episomal plasmid DNA. Results are the mean of four independent experiments ± standard deviation. The number of total colonies is indicated in parentheses. Statistical analysis was performed using Student’s *t*- test. *p = 0.0048 *vs***ITR**-*lys2URA3lys2*-**ITR**.

### The expression of Rep proteins increased AAV integration of a non-homologous fragment carrying ITRs

**Figure 4:**
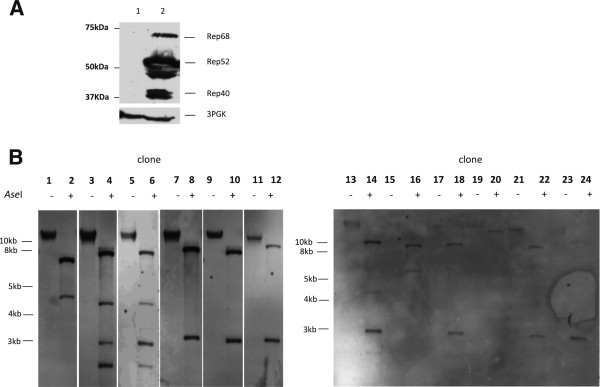
**Expression of Rep proteins and random integration of an AAV vector without homology with the yeast genome. A)** Western blot analysis of total cell lysate from yeast cells not expressing (lane 1) and expressing Rep proteins (lane 2) and transformed with pAAVPokURA carrying the ITRs. PGK3 antibody is used as loading control. **B)** Southern blot of genomic DNA of clones derived from transformation of Rep expressing yeast strain with pAAVPokURA. Lanes 1, 3, 5, 7, 9, 11, 13, 15, 17, 19, 21, 23: genomic DNA not restricted with *Ase*I; lane 2, 4, 6, 8, 10, 12, 14, 16, 18, 20, 22, 24: genomic DNA digested with *Ase*I that does not cut in the sequence between the ITRs containing Pok and URA3.

As Rep68 produces a site-specific ssDNA nick in the ITR, we thought that this ssDNA nick could affect both gene targeting and random integration. We used a yeast strain stably expressing all the Rep proteins to determine the effect on both gene targeting and random integration of the ITRs carrying fragment. The expression of Rep proteins is shown in the Western blot in Figure 4A. When the integration fragment shared homology with the genomic target (*LYS2* chromosomal gene), both gene targeting and random integration were not affected by the expression of Rep proteins (Table 2). However, when there is no homology between the fragment and the genome locus (ITR-*pok*URA-ITR, Figure 1A), the Rep expression significantly increased random integration (see Table 2). Presumably, the ssDNA nick at the level of ITR may be repaired through non-homologous recombination, resulting in an increase in random integration of the vector. Southern blot analysis of the genomic DNA extracted from a total of twelve clones derived from transformation of the yeast strain stably expressing Rep proteins was performed to check the number of stably integrated plasmid copies. It is interesting to note that eleven out of twelve clones contained at least two copies of the fragment (Figure 4B, lanes 2, 4, 6, 8 10, 12, 14, 16, 22, 24). This may be important for gene therapy application, where it is preferable to avoid vector random integration in order to prevent rearrangements.

**Table 2:** Effect of the AAV Rep protein expression on gene targeting and random integration in yeast

**Plasmid**	**Gene targeting**	**Random integration**	**GT/RI**	
**ITR**-*lys2URA3lys2*-**ITR, No REP**	31.8 ± 9.8	0.73 ± 0.39	43.5	
**ITR**-*lys2URA3lys2*-**ITR, REP**	20.1 ± 5.9	0.88 ± 0.49	22.8	
**ITR**-*pokURA3*-**ITR, No REP**	ND	0.12 ± 0.08	**/**	
**ITR**-*pokURA3*-**ITR, REP**	ND	1.02 ± 0.56*	**/**	

Yeast was transformed with *Pvu*II-restricted pAAVLUL or pAAVpokURA as reported in Materials and Methods. In the case of experiments with pAAVLUL, gene targeting and random integration events are reported as number of *URA3*^*+*^*lys2*^*-*^colonies /10^3^ transformants per μg of plasmid DNA and *URA3*^*+*^*LYS2*^*+*^ colonies /10^3 ^transformants per μg of plasmid DNA, respectively. Similarly, in the case of pAAVpokURA experiments, non-homologous integration events are reported as total *URA3*^*+*^colonies /10^3^ transformants per μg DNA. The number of transformants per μg of plasmid DNA was determined by transformation with 1 μg of episomal plasmid DNA. Results are the mean of four independent experiments ± standard deviation. The number of total colonies is indicated in the parentheses. Statistics was performed using Student’s *t*-test. *p = 0.0078 *vs***ITR**-*pokURA3*-**ITR, No REP.** ND = not detectable.

## Conclusions

Our results indicate that the ITRs and Rep proteins may affect AAV genomic random integration when no homology between the vector and the genome is present, and that rearrangements occur. Conversely, the presence of the AAV ITRs at both ends in a gene targeting construct sharing homology with a genomic locus could have an impact on the application of this strategy by significantly decreasing random integration.

## Methods

### Plasmids

The construction of the plasmid pAAVPokURA, which contains the URA3 marker and the 2.5 kb Pok stuffer sequence to increase the distance between the ITRs, has been previously reported [20] (Figure 1A). The plasmid pAAVLUL carrying the gene-targeting fragment between the two ITRs (Figure 1B), was constructed as follows: the 2.7 kb *Eco*RI-*Bam*HI fragment from pJZ102 [25] was first cloned into the pMCSsub; then, the entire fragment was cut off from pMCSsub with *Xba*I and inserted directly into the *Xba*I site of pSub201 containing the ITRs [20].

### Yeast transformation and molecular analysis

The yeast strain RSY12 (*MATa leu2–3,112 his3–11,15 URA3::HIS3*) has a complete deletion of the *URA3* gene, which was replaced with the *HIS3* gene [38]. To evaluate the effect of Rep proteins expression on gene targeting and random integration, we transformed the pAAV vectors in the RSY12 yeast strain containing the vector pG.Rep68 integrated into the genome [20].

Complete (YPAD) and synthetic complete (SC) medium were prepared according to standard procedures. Yeast was transformed with 3–5 μg of plasmid DNA using the standard lithium chloride method with single-stranded DNA as carrier [39]. The vectors pAAVLUL and pAAVpokURA were transformed, digested with either *Pvu*II or *Xba*I. Transformants were selected on SC-uracil (SC-URA). As the gene targeting events disrupt the chromosomal LYS2 gene by inserting the URA3 marker, the URA3 transformants were replica-plated in SC-lysine-uracil (SC-LYS -URA) plates to score for the random integration and gene targeting. In parallel, the same yeast culture was transformed with an episomal plasmid to evaluate the efficiency of transformation per μg of plasmid DNA. The frequency of gene targeting and random integration, calculated by dividing the number of events by the transformation efficiency, was expressed as number of URA^+^lys2^-^/10^3^ and URA^+^LYS2^+^/10^3^ total transformants per μg of plasmid DNA, respectively. Data were statistically evaluated by Student’s *t*-test with computer assistance. Single clones were grown in 5–10 ml of SC-URA and the genomic DNA isolated using the Master pure yeast DNA purification Kit (Epicentre Biotechnologies). The DNA was digested with *Ase*I, which does not cut in the rAAV fragment, electrophoresed, transferred to a nylon membrane (Roche), and hybridized with DIG-labelled -URA3 and ITR probes according to the standard protocol. DIG-labelled URA3 and ITR were obtained as previously described [20].

Western blot was performed with the monoclonal antibody 303.9 (PROGEN, Germany) and anti-3PGK (Invitrogen) as previously described [20].

To clone the junction sites, the genomic DNA from URA3^+^LYS2^+^ clones was digested with *Sph*I and ligated in the *Sph*I site of YEplac181 [40]. Then, the ligation mixture was transformed into competent *E.coli.* Single ampicillin-resistant colonies were grown in selection liquid medium, plasmid DNA extracted and sequenced with these primers: LYS2-UP 5’ TCCACTGCCAAGTATAGAA 3’ and LYS2-LOW 5’GTCATGTGGTAACACTGAA 3’.

## Competing interests

The authors declare no competing interests.

## Authors’ contributions

AG and TC conceived of the study and participated in its design and coordination. TC carried out the experimental work and analyzed the data. AG had a major contribution in writing the manuscript. Both authors read and approved the final manuscript.
